# SABRE Ir-IMes Catalysis for the Masses †

**DOI:** 10.3390/molecules30183837

**Published:** 2025-09-22

**Authors:** Izabelle Smith, Noah Terkildsen, Zachary Bender, Abubakar Abdurraheem, Shiraz Nantogma, Anna Samoilenko, Joseph Gyesi, Larisa M. Kovtunova, Oleg G. Salnikov, Igor V. Koptyug, Raphael Kircher, Danila A. Barskiy, Eduard Y. Chekmenev, Roman V. Shchepin

**Affiliations:** 1 Department of Chemistry Biology and Health Sciences, South Dakota School of Mines & Technology, Rapid City, SD 57701, USA; iodobromos@gmail.com (I.S.); noah.terkildsen@mines.sdsmt.edu (N.T.); thunder04298@gmail.com (Z.B.); 2 Department of Nanoscience and Engineering, Department of Chemistry Biology and Health Sciences, South Dakota School of Mines & Technology, Rapid City, SD 57701, USA; 3 Department of Chemistry, Integrative Biosciences (Ibio), Karmanos Cancer Institute (KCI), Wayne State University, Detroit, MI 48202, USA; abubakar@wayne.edu (A.A.); nantogmashiraz@wayne.edu (S.N.); annasam1512@wayne.edu (A.S.); jngyesi@wayne.edu (J.G.); chekmenev@wayne.edu (E.Y.C.); 4 International Tomography Center SB RAS, Novosibirsk 630090, Russia; kovtunova@catalysis.ru (L.M.K.); salnikov@tomo.nsc.ru (O.G.S.); koptyug@tomo.nsc.ru (I.V.K.); 5 Institute of Physics, Helmholtz Institute Mainz, Johannes Gutenberg Universität Mainz, 55099 Mainz, Germany; rkircher@uni-mainz.de; 6 Frost Institute for Chemistry and Molecular Science, Department of Chemistry, University of Miami, Coral Gables, FL 33146, USA; barskiy@miami.edu

**Keywords:** hyperpolarization, parahydrogen, SABRE, NMR spectroscopy, iridium

## Abstract

The Signal Amplification By Reversible Exchange (SABRE) technique provides enhancement of Nuclear Magnetic Resonance (NMR) signals up to several orders of magnitude using chemical exchange of a substrate and parahydrogen on an iridium complex. Therefore, the availability of such a catalytic complex to a broader community is an absolutely vital step for dissemination of the groundbreaking SABRE methodology. The most common SABRE catalyst, which is activated in situ, is based on Ir-IMes system (IMes = 1,3-Bis(2,4,6-trimethylphenyl)imidazol-2-ylidene). Earlier approaches for the synthesis of this catalyst often relied on specialized equipment and were limited to a comparatively small scale. This, in turn, increased the barrier of entry for new scientists to the area of SABRE hyperpolarization. Here, we present a robust, inexpensive, and easy to reproduce synthetic procedure for the preparation of this SABRE catalyst, which does not require specialized inert atmosphere equipment like a glove box or Schlenk line. The synthesis was validated on the scale of several grams vs. tens of milligrams scale in the reported approaches. The resulting SABRE catalyst, [Ir(IMes)(COD)Cl], was activated in situ and further evaluated in hyperpolarization experiments resulting in signal enhancements comparable to (or higher than) those for the catalyst prepared using Schlenk line equipment.

## 1. Introduction

Nuclear magnetic resonance (NMR) spectroscopy and magnetic resonance imaging (MRI) techniques are used for a broad range of applications, such as structural characterization of chemicals and biomolecules, quantitative analysis, metabolomics, materials science, preclinical studies, and clinical diagnostics. However, the sensitivity of these techniques is inherently low, as the thermal polarization of nuclear spins is on the order of 10^−4^ to 10^−5^ for protons even at magnetic fields of modern NMR spectrometers and MRI scanners of several Tesla. Hyperpolarization techniques provide temporal enhancement of nuclear spin polarization of up to several orders of magnitude [1,2,3,4,5,6]. The main driver behind the development of hyperpolarization techniques is their prospective clinical applications [3,7,8,9,10,11,12,13,14]. In particular, hyperpolarized (HP) [1-^13^C]pyruvate has shown its utility as a molecular contrast agent for MRI diagnostics of abnormal metabolism, including cancerous tumors [7,8,15]. For solution-state hyperpolarization of molecules, the most efficient and convenient hyperpolarization techniques are dissolution dynamic nuclear polarization (dDNP) [16,17,18], and two parahydrogen-based approaches: parahydrogen-induced polarization (PHIP) [19,20,21,22] and signal amplification by reversible exchange (SABRE) [23,24,25,26].

**Figure 1:**
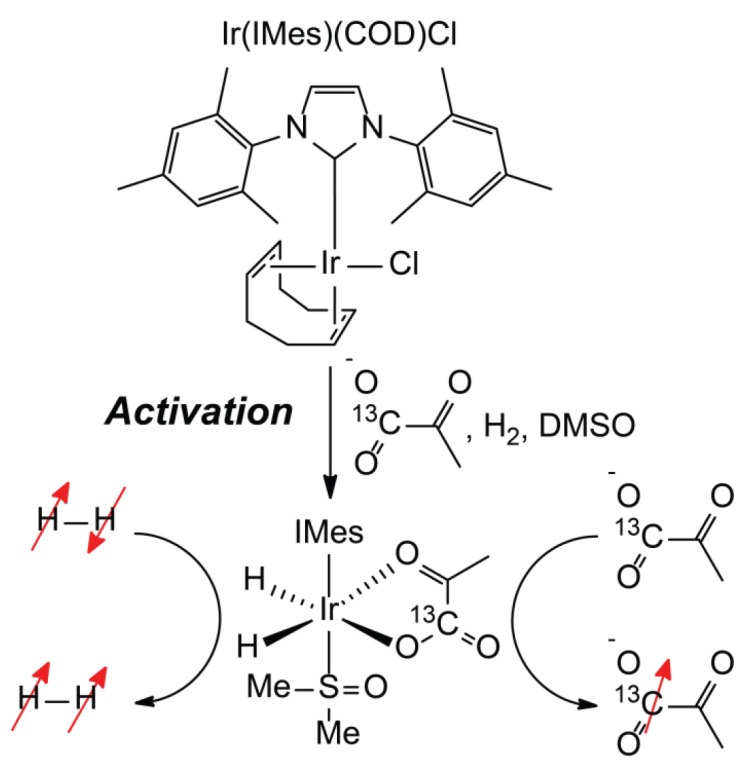
Scheme of the SABRE catalyst activation and the hyperpolarization process shown on the example of [1-^13^C]pyruvate.

In the pursuit of affordable hyperpolarization techniques, PHIP and SABRE stand out as they operate without the need for strong magnetic fields, cryogenic temperatures of several kelvin, or high-frequency microwaves to generate strong signals on heteronuclei [1]. PHIP and SABRE utilize parahydrogen (*p*H_2_, a nuclear spin isomer of H_2_ with the total nuclear spin *I* = 0) as a source of nuclear spin order. Sophisticated and expensive, devices based on the use of closed-cycle helium cryocompressors can generate nearly 100% *p*H_2_ [27,28,29]. This is excellent for medical applications, but may not be critical for research studies. On the other hand, the entrance barrier to the area of *p*H_2_-based hyperpolarization for the broader science community can be drastically decreased via the use of straightforward and inexpensive 50% *p*H_2_ generators (providing only a 3-fold lower signal enhancement compared to 100% *p*H_2_—not a dramatic difference considering that signal enhancement factors on the order of 10^3^ to 10^5^ can be achieved at magnetic fields of several Tesla [30,31,32]) based on a tube loaded with FeO(OH) ortho-para conversion catalyst immersed in liquid nitrogen [33,34]. After enrichment, *p*H_2_ gas can be stored for several weeks if the storage tank has no paramagnetic impurities [35]. In PHIP, *p*H_2_ is catalytically added to a double or a triple carbon–carbon bond in a pairwise manner, i.e., with the two H atoms from the same *p*H_2_ molecule ending up in the same reaction product, retaining the nuclear spin correlation between them [36]. In SABRE, *p*H_2_ and a substrate reversibly coordinate to a metal complex (Figure 1). Spin order is transferred from H nuclei originating from *p*H_2_ to substrate nuclei within the complex, and subsequent chemical exchange results in the accumulation of hyperpolarized substrate molecules in solution [37]. Thus, unlike in PHIP, substrates in SABRE are not chemically modified during the hyperpolarization process. The SABRE process was recently utilized for testing a low-cost hyperpolarization system, which negates the need for an expensive instrument, further reducing the barrier to broader adoption of SABRE methodology [38].

The remaining barrier for SABRE methodology is the availability of a suitable polarization transfer catalyst (PTC) for efficient spin order transfer. A typical SABRE catalyst comprises an Ir complex, [Ir(NHC)(COD)Cl], where NHC is an N-heterocyclic carbene ligand [39]. In the presence of a substrate (S) and hydrogen, depending on the choice of solvent, the active SABRE PTC [Ir(NHC)(H)_2_(S)_3_]^+^ or [Ir(NHC)(H)_2_(S)_2_Cl] is formed [40,41], which then participates in substrate and *p*H_2_ exchange, facilitating polarization transfer and accumulation of HP substrate (Figure 1). While, in principle, the catalyst structure needs to be adjusted for each substrate individually [42,43], [Ir(IMes)(COD)Cl] (IMes = 1,3-Bis(2,4,6-trimethylphenyl)imidazol-2-ylidene) complex appears to be a nearly universal choice of the catalyst, i.e., it typically provides the highest NMR signal enhancements [31,44,45,46].

**Figure 2:**
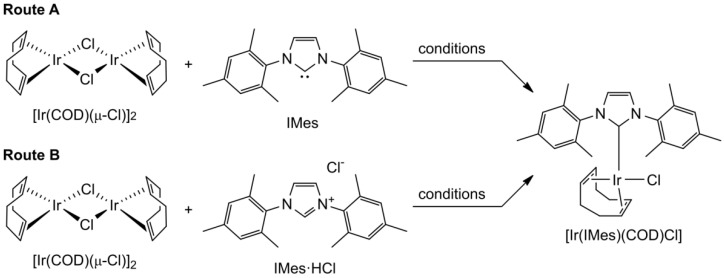
Scheme of the reported routes of [Ir(IMes)(COD)Cl] SABRE catalyst synthesis. The corresponding reaction conditions and yields are presented in Table 1.

Of note, [Ir(IMes)(COD)Cl] SABRE catalyst is currently not readily commercially available and needs to be synthesized on demand, typically from [Ir(COD)(μ-Cl)]_2_ dimer and IMes ligand (Figure 2, Route A) [47,48]. It is worth pointing out that while commercially available Crabtree’s catalyst [Ir(PCy_3_)(COD)(Py)]^+^ has been employed in the early SABRE studies [49], its performance is inferior to that of [Ir(IMes)(COD)Cl] [39]. The presence of the phosphine ligand introduces additional ^1^H-^31^P couplings, which split the single maximum in the SABRE magnetic field profile into two, thereby reducing overall polarization efficiency [50]. By contrast, the Ir–IMes system has emerged as the established benchmark for SABRE catalysis, enabling higher levels of spin polarization and broad utility across substrates [31]. For this reason, current methodological work—including the present study—focuses on Ir–IMes as the catalyst of choice rather than Crabtree’s catalyst.

**Table 1:** Summary of the reported syntheses of [Ir(IMes)(COD)Cl] SABRE catalyst.

Route	Conditions	Yield (%)	Reference
A	THF, RT, inert atmosphere	52	[47]
A	benzene, RT, inert atmosphere	93	[48]
B	^t^BuOK, THF, RT, inert atmosphere	64	[45]
B	K_2_CO_3_, acetone, 60 °C, air	67	[51]
A	benzene, RT, air	82	[52]
B	K_2_CO_3_, acetone, 60 °C, air	85	[52]

The originally proposed synthetic protocols for the preparation of [Ir(IMes)(COD)Cl] SABRE catalyst required performing the entire synthetic process under an inert atmosphere using either the Schlenk technique or a glove box; solvents should be dried and distilled under an inert atmosphere as well. Later, it was proposed to replace IMes with its corresponding hydrochloride (IMes·HCl) and to add K_2_CO_3_ as a base (Figure 2, Route B) [45,51]. In this case, it was possible to avoid the requirement of an inert atmosphere and perform all procedures under air and with technical grade solvents, although the reaction was carried out for 20 h at 60 °C [51]. Next, Blanchard et al. found that the reaction of [Ir(COD)(μ-Cl)]_2_ and IMes can be successfully performed at ambient atmosphere, and a high yield of 82% can be achieved in just 5 min [52]. Despite the efficiency of this synthetic protocol, there is still room for improvement. In particular, the reported procedures were performed on an extremely small scale (mg) in pressure vials with special equipment for their agitation.

This work addresses this remaining challenge by providing an easy-to-follow large-scale synthesis of the [Ir(IMes)(COD)Cl] catalyst typically employed in the SABRE process. The presented synthetic protocol does not demand specialized equipment and is based on a less expensive protonated IMes·HCl ligand. Due to simplicity, it is suitable for a greater science community outside of synthetic chemistry circles. The SABRE performance of the prepared material was shown to be similar to that of [Ir(IMes)(COD)Cl] synthesized using literature protocols [48].

## 2. Results and Discussion

IMes·HCl hydrochloride is typically ca. 2 times less expensive than the neutral IMes ligand. More importantly, “Route B” does not require a glove box, and it is tolerant to commercial solvents and atmospheric conditions (to some extent). Therefore, we set ourselves to optimize “Route B” approach for the affordable large-scale synthesis of [Ir(IMes)(COD)Cl]. Our initial inclination was to simply scale up the synthesis described in the references [51,52]. However, all the attempts to conduct the preparation procedure similar to that of “Route B” (Figure 2) at acetone boiling temperature (~56 °C) using a reflux condenser resulted in the prevalence of polymerization products, rendering the method impractical (Table 2, Entry 1). The exact reason for the prevalence of side reactions (even under an argon atmosphere) when the reaction was scaled from 60 mg to several grams remains to be found. However, we can speculate that the difference in mass transfer of the starting materials that are only partially soluble in acetone played a significant role in this outcome. Thus, the initial strategy had to be revised.

It is well known that the rate of an average chemical reaction expedites ~2 times with every 10 °C reaction temperature increase. The reverse of this empirical law is true as well. Thus, we decided to conduct the reaction at room temperature, drastically simplifying the synthetic setup used in the process. Consequentially, such simplification came at the expense of comparatively long reaction time. Thus, the reaction was conducted in acetone at the typical scale based on Ir starting material (4.0 g of [Ir(COD)(μ-Cl)]_2_) over 8 days (Table 2, Entry 2). The progress of the reaction was monitored using the ^1^H-NMR signal of the protonated ligand at 10.33 ppm, which fully disappears upon reaction completion (Figure S1). It is worth pointing out that the monitoring of the reaction was performed based on a relatively affordable 60 MHz (^1^H frequency) cryogen-free benchtop NMR spectrometer, making it accessible to the broader science community. 

Next, we further experimented with reaction time and reagent concentrations. A longer reaction time seemingly resulted in a slightly higher overall yield (Table 2, Entry 3). An increase in concentrations of both reactants resulted in an apparent increase in the reaction rate (Table 2, Entry 4), which is not surprising considering the expected kinetics of the reaction. However, the scaled-up reaction resulted in a lower yield and a somewhat larger amount of visible polymerization products. While we could not detect by NMR the presence of side products in catalyst batches prepared by the methods described above (Table 2, Entries 2–4), the resulting catalyst had somewhat amorphous structure and “muddy” yellow-red color (Figure S2) even after preparative chromatography using gravity silica as a stationary phase and dichloromethane (DCM) as an eluent. It is also worth pointing out that recrystallization of the final product from pentane described in the literature [51,52] did not work on the multigram scale in our experience. Therefore, we decided to investigate other solvents as the reaction medium. DCM was briefly evaluated. However, it was found to be reactive with IMes·HCl even in the absence of the iridium precursor [Ir(COD)(μ-Cl)]_2_ (Figure S3).

Acetonitrile was the next obvious choice of solvent stable under the reaction conditions. It is expected because acetonitrile is an aprotic solvent with an even higher dielectric constant compared to acetone (36.6 vs. ~21 for acetone). Thus, we expected that acetonitrile would allow us to conduct the preparation process in a manner similar to the acetone-based procedure described above. First, acetonitrile was used under standard conditions (Table 2, Entry 5) similar to those of Table 2, Entry 3. The reaction was completed notably faster than that in the case of acetone (Table 2, Entry 3). Despite the significant decrease in the nominal reaction yield, the crude reaction mixture had a clean yellow color (Figure S4), indicating an apparent decrease in the amount of polymerized (dark) side products compared to the synthesis in acetone (photo not shown). Purification of the product was conducted, as before, via column chromatography (silica gel and DCM as an eluent, Figure S5), resulting in a visibly cleaner separation (Figure S6) than that for the previous acetone-based synthesis (photo not shown). More importantly, however, the final product appeared both cleaner and more crystalline (Figure S6) than that obtained (Figure S2) from reaction in acetone (Table 2, Entries 2–3). Therefore, all further reactions were conducted in acetonitrile media.

Afterward, we experimented with the slight excess of the iridium precursor, [Ir(COD)(μ-Cl)]_2_. Initially, we planned to increase the reaction duration. However, we noticed the persistent presence of the IMes·HCl peak at 10.33 ppm in the ^1^H-NMR spectrum even after several days of reaction. Addition of [Ir(COD)(μ-Cl)]_2_ allowed us to rapidly (<24 h) complete both reactions and to increase their overall yield (Table 2, Entries 6–7). There can be two (not mutually exclusive) explanations. First, the formation of the desired product competes with the decomposition of [Ir(COD)(μ-Cl)]_2_. Thus, an additional iridium starting material infusion was required to achieve full conversion of IMes·HCl. Also, it is not implausible that some batches of [Ir(COD)(μ-Cl)]_2_, commercial starting material, had somewhat lower purity, leading to IMes·HCl being in some excess to iridium. Due to the requirement of producing large amounts of the final product, the procedure was scaled up proportionally by fourfold (Table 2, Entry 8). No extra amounts of Ir starting material were used, resulting in a somewhat lower but acceptable overall yield. During the course of this study, it came to our attention that environmental safety agencies of both the EU and the US are planning to significantly restrict the usage of dichloromethane (DCM) even in research settings. Because our procedure relied on significant amounts of DCM for purification of the final product, DCM was replaced by acetonitrile as an eluent in the next synthesis entry (Table 2, Entry 9). The yield and the crystal appearance were consistent with the previous experiment (Table 2, Entry 8). However, acetonitrile (at least in its pure form) was found to be a less-than-ideal eluent for this process. Approximately 5–7 times larger amounts of acetonitrile had to be used in order to separate the final product. Further experiments, which may include the mixture of acetonitrile with more polar solvents (e.g., alcohols) can be evaluated in the future in order to effectively replace DCM. Here, we show that, in principle, DCM-free version of the procedure on a large scale is possible if DCM application becomes unfeasible due to the ecologically driven regulations. To conclude the synthetic part of this section, the importance of flashing the reaction flask with argon was evaluated (Table 2, Entry 10). In contrast to the previous reports [51], which suggested that the use of an inert gas is not required, in our experiments, omission of such an argon flash resulted in a significantly diminished product yield. Therefore, such an omission is not recommended unless argon gas is not available at the site of catalyst preparation. In total, more than 50 g of the final product was prepared as a result of the procedures described above (Table 2, Entries 2–10).

**Figure 3:**
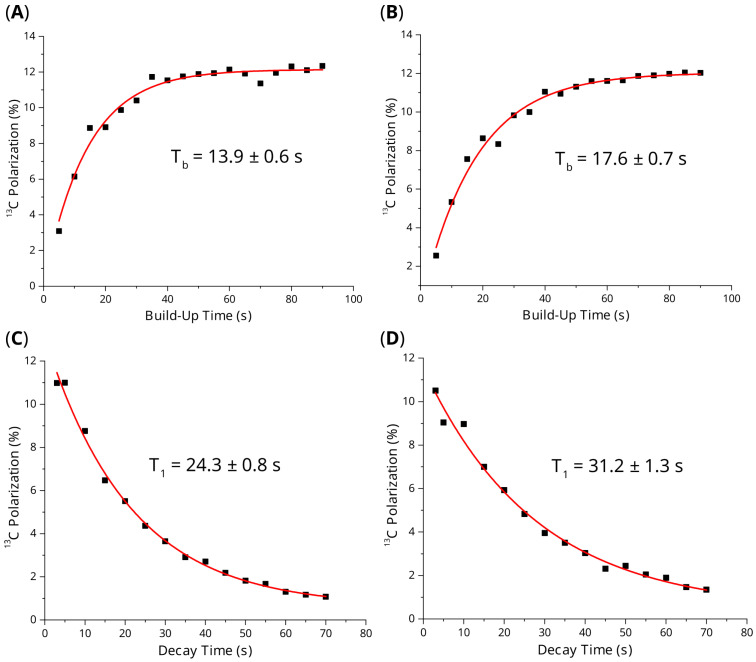
Kinetics of ^13^C SABRE-SHEATH polarization buildup and decay of [1-^13^C]pyruvate at 0.4 μT magnetic field and 6 °C. (**A**) Polarization buildup measured using the SABRE catalyst synthesized via the procedure described in this work. (**B**) Polarization buildup measured using the SABRE catalyst synthesized via the procedure from Ref. [48]. (**C**) Polarization decay measured using the SABRE catalyst synthesized via the procedure described in this work. (**D**) Polarization decay measured using the SABRE catalyst synthesized via the procedure from Ref. [48].

The performance of the synthesized SABRE catalyst (a combined mixture of all fractions, Table 2, Entries 2–10) was evaluated in comparison with the material prepared using the standard Schlenk line approach [48]. Therefore, SABRE in SHield Enables Alignment Transfer To Heteronuclei (SABRE-SHEATH [53]) experiments with [1-^13^C]pyruvate were performed (Figure S7). To date, [1-^13^C]pyruvate is the most important tracer that can be hyperpolarized using dDNP [54,55,56], PHIP [57,58,59,60,61] and SABRE [62,63,64,65]. Pyruvate is an important metabolite, and injection of ^13^C HP [1-^13^C]pyruvate into an organism allows one to monitor metabolic transformations of pyruvate, reporting on various pathologies, including cancerous tumors [60,66,67,68,69]. The two comparative measurements of ^13^C-NMR polarization buildup and decay at a submicrotesla magnetic field were conducted (Figure 3). In the given study, our catalyst performed slightly better, resulting in ~12.3% ^13^C polarization level (>100,000-fold signal enhancement at 1.4 T field, Figure 3A, Table S1; here and below, total polarization for sum of free and Ir-bound forms of [1-^13^C]pyruvate is presented) vs. ~12% for the catalyst prepared by the literature’s procedure [48] (Figure 3B, Table S2). These polarization levels compare well with the previous literature data [65]. We have also noticed that polarization buildup (*T*_b_) and decay (*T*_1_) for the catalyst synthesized using the approach reported in this work (Figure 3A,C, Tables S1 and S3) were slightly faster than that for the catalyst prepared using the literature procedure [48] (Figure 3B,D, Tables S2 and S4). This difference can be tentatively attributed to the different proportion of paramagnetic impurities that are generated during the synthesis. Overall, the catalyst prepared in this study demonstrated similar performance to that of the material prepared using the literature protocol [48].

## 3. Materials and Methods

Dichloromethane and bis(1,5-cyclooctadiene)diiridium(I) dichloride were obtained from Sigma-Aldrich (St. Louis, MO, USA). Acetone, acetonitrile, and chloroform-D with TMS were procured from Thermo Fisher Scientific (Waltham, MA, USA). 1,3-bis(2,4,6-trimethylphenyl)imidazolium chloride was purchased from STREM Chemicals (Newburyport, MA, USA). Silica Flash was purchased from Silicycle (Quebec City, QC, Canada). NMR analysis was conducted on a 60 MHz ^1^H spectrometer (Nanalysis Corp., Calgary, AB, Canada).

### 3.1. General Synthesis Procedure

**Figure 4:**
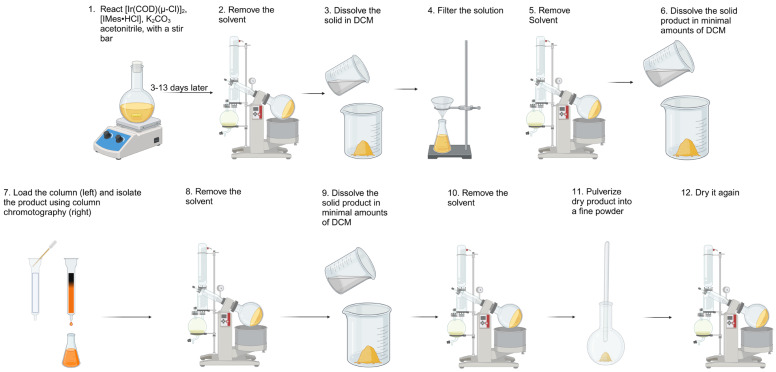
Schematics of the SABRE catalyst step-by-step preparation procedure intended for the broader scientific community.

In order to better illustrate the entire preparation process for the broader scientific community, each step of the process is shown schematically in Figure 4. A meticulously cleaned 500 mL round bottom flask (RBF) was placed onto a stir plate and flushed with argon gas for 5 min. After that, bis(1,5-cyclooctadiene)diiridium(I) dichloride (4.000 g, 1.0 eq, 5.955 mmol), bis(2,4,6-trimethylphenyl)imidazolium chloride (4.060 g, 2.0 eq., 11.91 mmol), and potassium carbonate (4.930 g, 6.0 eq., 35.73 mmol) were added to the RBF. Once the addition of the solids was completed, 200 mL of solvent (acetone or acetonitrile, see Table 2) was bubbled with argon gas, and poured into the RBF, followed by the addition of a 1.5-inch stir bar. The final mixture within the RBF was flushed with argon once more, and the stirring was set to 350 rotations per minute (Figure 4, step #1). To protect the reaction from light, the RBF was wrapped in aluminum foil. The reaction solution was allowed to mix for a period of 3–13 days, while regular 0.5 mL samples were withdrawn for NMR analysis to track the reaction progress (Figure 4, step #1). Upon completion of the reaction, the RBF was removed from the stir plate, and the neck was thoroughly cleaned with solvent and a Kimwipe^®^ to ensure no contaminants were introduced. Similarly, the rotational evaporator neck and bump trap were cleaned with solvent to ensure an airtight seal was obtained. The solution was dried with the use of a rotational evaporator, gradually decreasing the pressure and increasing the spin rate over the course of 20–30 min until the system’s pressure reached ca. 200 mbar. Then pressure gradually decreased to 1 mbar at a constant spin rate. Note: releasing the pressure and emptying the solvent trap of the rotational evaporator may be needed in order to reach the desired 1 mbar pressure. This pressure was kept for a minimum of 1 h (Figure 4, step #2). Following drying, a gravity filtration setup was prepared, and the dried material was dissolved in minimal amounts of DCM (Figure 4, step #3). The solution was then gravity-filtered to remove inorganic salts. From there, the RBF and the funnel were rinsed with additional DCM as needed to ensure complete transfer of the product solution (Figure 4, step #4). The neck of the RBF was cleaned with DCM and a Kimwipe^®^, and the solution was subjected to further drying on the rotational evaporator (as described above) until complete dryness (Figure 4, step #5). After that, a 250 mL chromatography column was prepared by plugging the bottom of the column with a small piece of cotton before packing it with a silica gel slurry comprising ~100 mL silica gel and 80 mL solvent (DCM or acetonitrile, see Table 2). The product was dissolved (Figure 4, step #6) in the same solvent as the column’s eluent and carefully transferred to the column (Figure 4, step #7). The stopcock was opened to release the solvent until the solution reached the surface of the silica, after which clean solvent was added to the top of the column to begin the separation. This process was repeated multiple times to ensure complete loading of the product solution into the silica gel. Once loaded, the column was covered with a layer of solvent, and the product solution was collected as it eluted from the column as an orange solution (Figure S5). The collected solution was put into a rotational evaporator inside a 1 L RBF to evaporate (as above) most of the solvent. This was followed by further drying at 1 mbar pressure for 1 h (Figure 4, step #8). The remaining solid was dissolved (Figure 4, step #9) in the minimal amount of solvent (DCM or acetonitrile, see Table 2) and transferred to a smaller RBF for final drying using the rotational evaporator (Figure 4, step #10). The resulting solid was crushed using a stir rod (Figure 4, step #11) and dried again using the rotational evaporator. Note: keep rotation to the minimal setting. The solid product was then transferred to a vial for subsequent use. The final product is a bright yellow powder. For long-term storage (years), no special precautions are needed and the synthesized material can be stored at ambient atmosphere in a refrigerator or at room temperature. The general synthesis procedure described above was modified for some of the synthesis entries, as outlined in Table 2.

### 3.2. SABRE-SHEATH Hyperpolarization Experiments

[1-^13^C]pyruvate (6.6 mg) and DMSO (6.3 mg) were dissolved in a vial in 2 mL of methanol-d_4_ (1.8 g), resulting in [1-^13^C]pyruvate and DMSO concentrations of 30 mM and 40 mM, respectively. The vial was then vortexed until the solution components were fully mixed. Next, 0.6 mL of the prepared [1-^13^C]pyruvate/DMSO stock solution was added to the SABRE catalyst [Ir(IMes)(COD)Cl] (2.3 mg) in a vial. The solution was then vortexed until properly mixed and transferred into a Teflon-jacketed 5 mm NMR tube. Once transferred, the solution was bubbled with argon gas to remove dissolved oxygen. The NMR tube was connected to the SABRE setup described in detail elsewhere [70]. The sample was bubbled with parahydrogen gas for 10 min to activate the catalyst into the SABRE-active complex [Ir(IMes)(H)_2_(DMSO)(η_2_-[1-^13^C]pyruvate)]. Then the NMR tube was inserted into the magnetic shield (0.4 μT field) and the sample was cooled down to 6 °C. *p*H_2_ gas was bubbled through the sample for 60 s at 8 bar pressure and 120 standard cubic centimeters per minute (sccm) gas flow rate. Then the sample was transferred to a SpinSolve Carbon 60 (Magritek, New Zealand) benchtop NMR spectrometer, and a ^13^C-NMR spectrum was acquired with a 90° RF pulse. To measure polarization buildup, the duration of *p*H_2_ bubbling was varied, while for the polarization decay measurements, the sample was kept inside the magnetic shield for a variable time after gas bubbling was terminated.

## 4. Conclusions

In this work, we present a new variant of the procedure for the preparation of SABRE catalyst [Ir(IMes)(COD)Cl] on a previously unprecedented scale, multigram per batch (50 g in total over ten batches). The developed approach is robust, and the synthesis procedure can be performed in practically any “wet” laboratory, which may lack advanced equipment such as a glove box or even access to inert gases. In addition, IMes·HCl ligand is significantly less expensive than its “free” form IMes, lowering the overall cost of the process. We also addressed the issue of ecological regulations by developing a DCM-free version of this synthetic methodology. While the methodology described here has some room for improvement in terms of yields, it drastically lowers the entry barrier to the area of NMR hyperpolarization for anyone wishing to join this exciting area of science.

## Figures and Tables

**Table 2:** Summary of the [Ir(IMes)(COD)Cl] syntheses performed in this study.

Entry	Solvent	Reaction Time (Days)	Yield (%)	Modifications to the General Procedure
1	Acetone	~0.8	0	~56 °C (acetone reflux)
2	Acetone	8	78	None
3	Acetone	11	81	None
4	Acetone	3	63	×2 concentrations compared to the general procedure
5	Acetonitrile	3	63	None
6	Acetonitrile	8	67	1.0 g of extra [Ir(COD)(μ-Cl)]_2_ added after 7 days
7	Acetonitrile	13	70	1.2 g of extra [Ir(COD)(μ-Cl)]_2_ added after 12 days
8	Acetonitrile	3	59	×4 concentrations compared to the general procedure
9	Acetonitrile	4	57	×4 concentrations compared to the general procedure; acetonitrile used for column solvent
10	Acetonitrile	8	45	no argon flushing

## Data Availability

Raw spectroscopic data is available at Zenodo: https://doi.org/10.5281/zenodo.17150107.

## Supplementary Materials

The following supporting information can be downloaded at: https://www.mdpi.com/article/10.3390/molecules30183837/s1, Figure S1: Representative spectrum set (^1^H-NMR, 60 MHz). The trace above (in red) presents an initial reaction aliquot showing a characteristic starting material (IMes·HCl) peak at 10.33 ppm. The trace below (blue) represents a completed reaction (with no IMes·HCl peak); Figure S2: Product of entry #3 (Table 2, main text); Figure S3: ^1^H-NMR spectra (60 MHz) showing reactivity of IMes in DCM without [Ir(COD)(μ-Cl)]_2_ addition. Red trace: pure IMes·HCl ligand in CDCl_3_. Blue trace: IMes·HCl + K_2_CO_3_ in DCM after 24 h; taken aliquot was evaporated on rotational evaporator and dissolved in CDCl_3_; Figure S4: Crude reaction mixture in MeCN (Table 2, entry #5); Figure S5: Stages of column chromatography (gravity silica gel and DCM as an eluent, Table 2, entry #5); Figure S6: Final product (Table 2, entry #5); Figure S7: ^13^C-NMR spectra of SABRE-SHEATH-hyperpolarized [1-^13^C]pyruvate (total concentration 30 mM, blue trace) and thermal reference spectrum of neat [1-^13^C]acetic acid (17.5 M, black trace, multiplied by a factor of 32); Table S1: [1-^13^C]pyruvate SABRE-SHEATH polarization buildup data for the catalyst synthesized in this work (data for Figure 3A); Table S2: [1-^13^C]pyruvate SABRE-SHEATH polarization buildup data for the catalyst made by literature procedure [48] (data for Figure 3B); Table S3: [1-^13^C]pyruvate SABRE-SHEATH polarization decay data for the catalyst synthesized in this work (data for Figure 3C); Table S4: [1-^13^C]pyruvate SABRE-SHEATH polarization decay data for the catalyst made by the literature procedure [48] (data for Figure 3D); Reference [48] is cited in the supplementary materials.
