# Supplementary material for: SABRE Ir-IMes Catalysis for the Masses
Source: Molecules. 2025 Sep 22;30(18):3837. doi: 10.3390/molecules30183837 (PMC12473097; doi:10.3390/molecules30183837)
Supplement: Supplementary file 1 [file molecules-30-03837-s001.zip › molecules-3872040-supplementary.pdf]

Supplementary Materials

SABRE Ir-IMes Catalysis for the Masses

Izabelle Smith, Noah Terkildsen, Zachary Bender, Abubakar Abdurraheem, Shiraz Nantogma, Anna Samoilenko, Joseph Gyesi, Larisa M. Kovtunova, Oleg G. Salnikov, Igor V. Koptyug, Raphael Kircher, Danila A. Barskiy, Eduard Y. Chekmenev, Roman V. Shchepin*

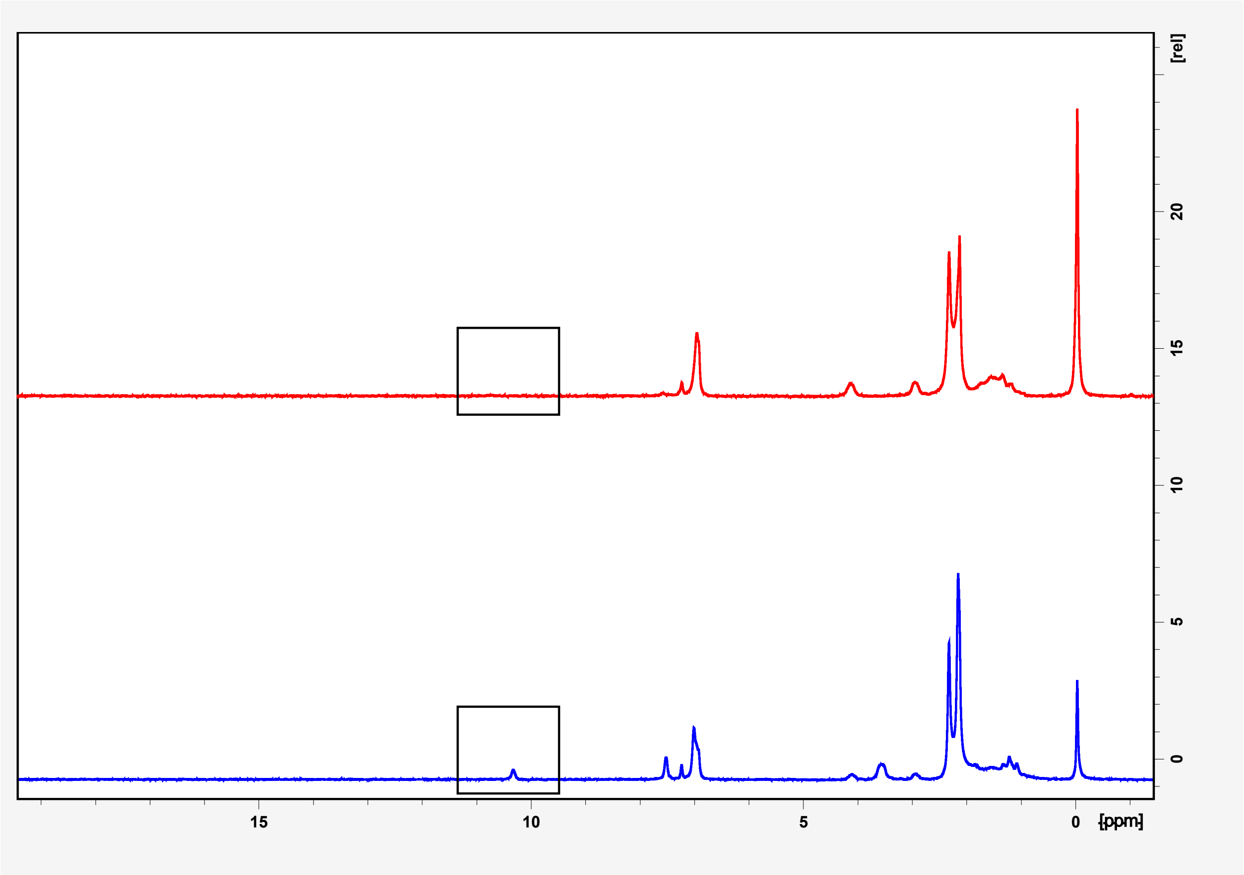

Figure S1. Representative spectrum set (^{1}H NMR, 60 MHz). The trace below (in blue) presents initial reaction aliquot showing characteristic starting material (IMes·HCl) peak at 10.33 ppm. The trace above (in red) represents completed reaction (with no IMes·HCl peak).

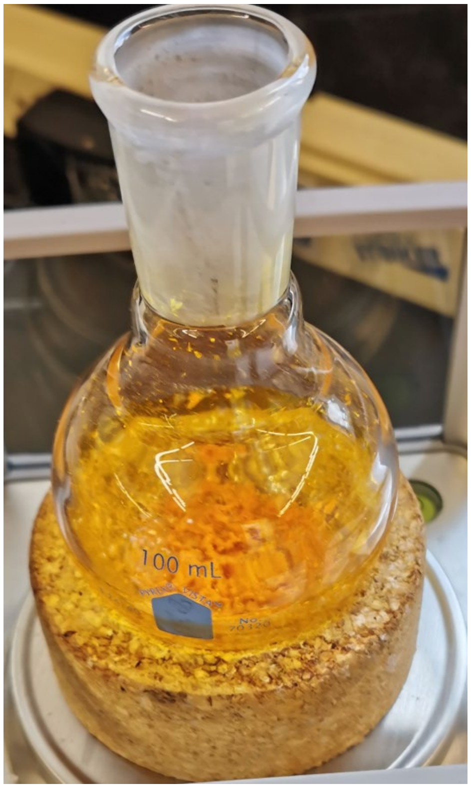

Figure S2. Product of entry #3 (Table 2, main text).

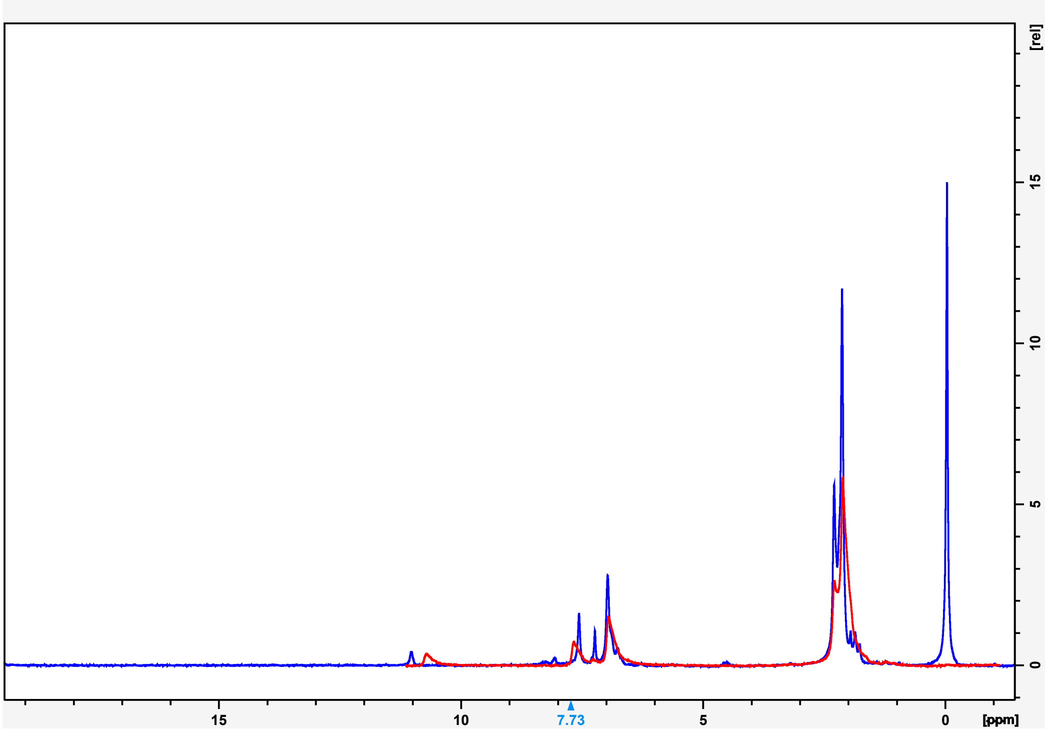

Figure S3. ^{1}H NMR spectra (60 MHz) showing reactivity of IMes in DCM without $[Ir(COD)(\mu\text{-}Cl)]_2$ addition. Red trace: pure IMes·HCl ligand in $CDCl_3$. Blue trace: IMes·HCl + K_2CO_3 in DCM after 24 hours; taken aliquot was evaporated on rotational evaporator and dissolved in $CDCl_3$.

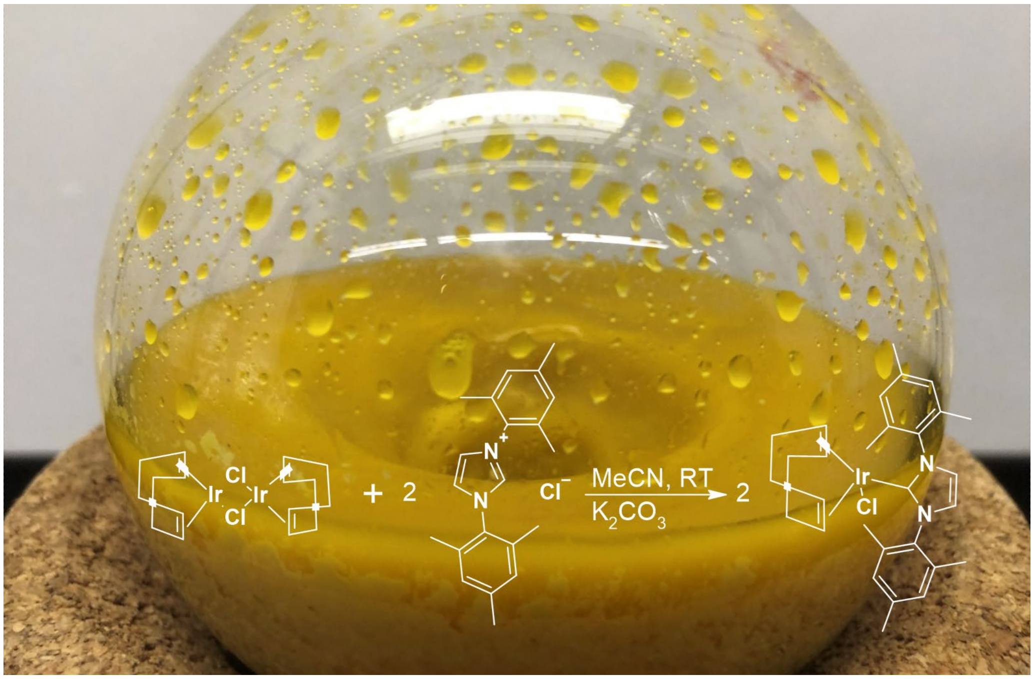

Figure S4. Crude reaction mixture in MeCN (Table 2, entry #5).

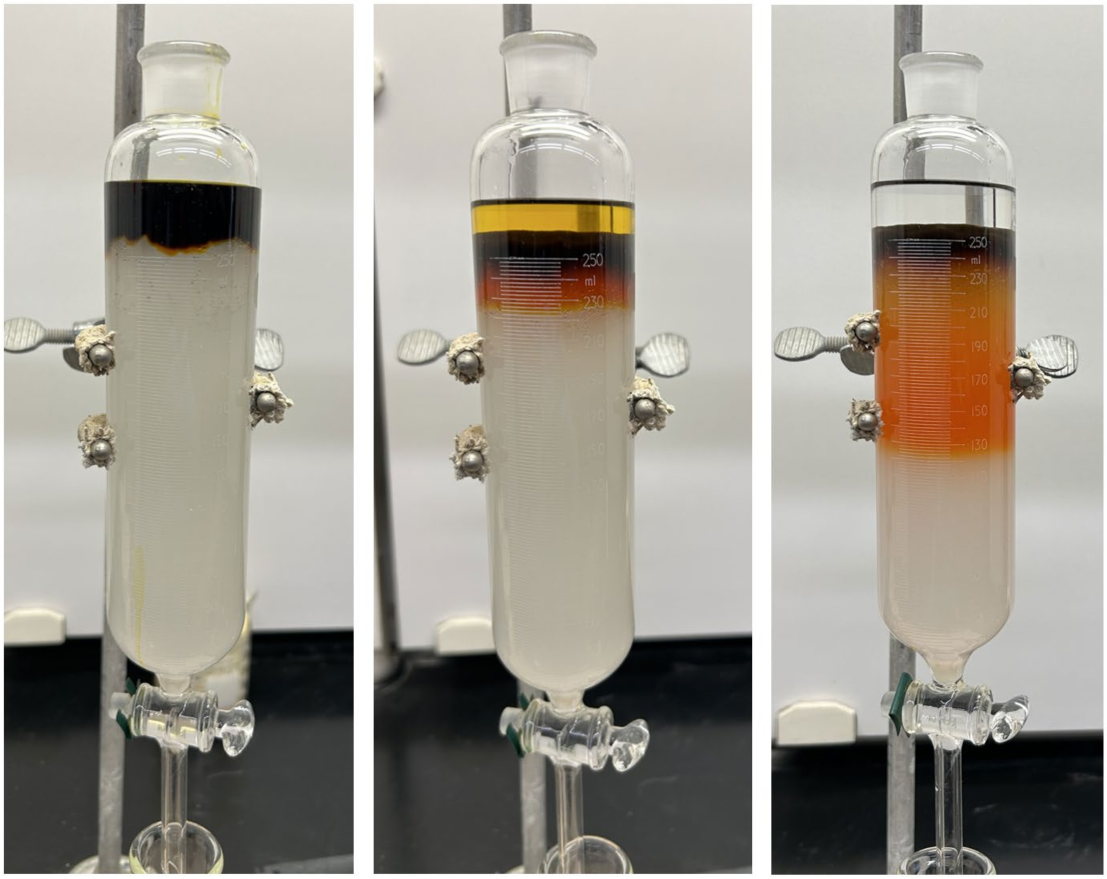

Figure S5. Stages of column chromatography (gravity silica gel and DCM as an eluent, Table 2, entry #5).

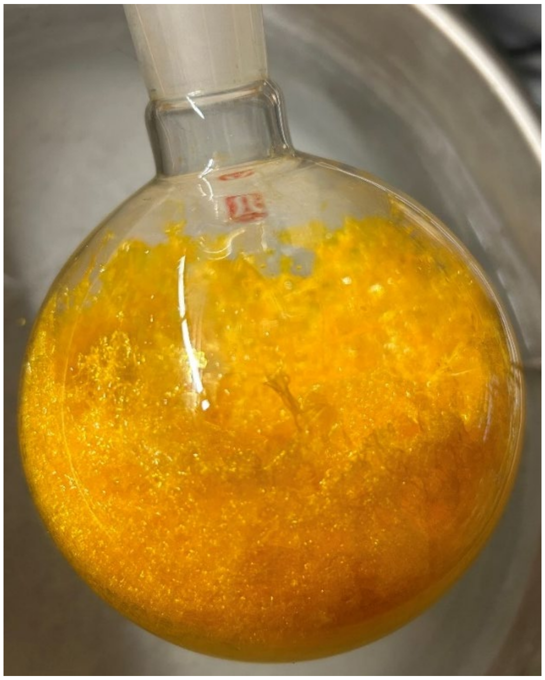

Figure S6. Final product (Table 2, entry #5).

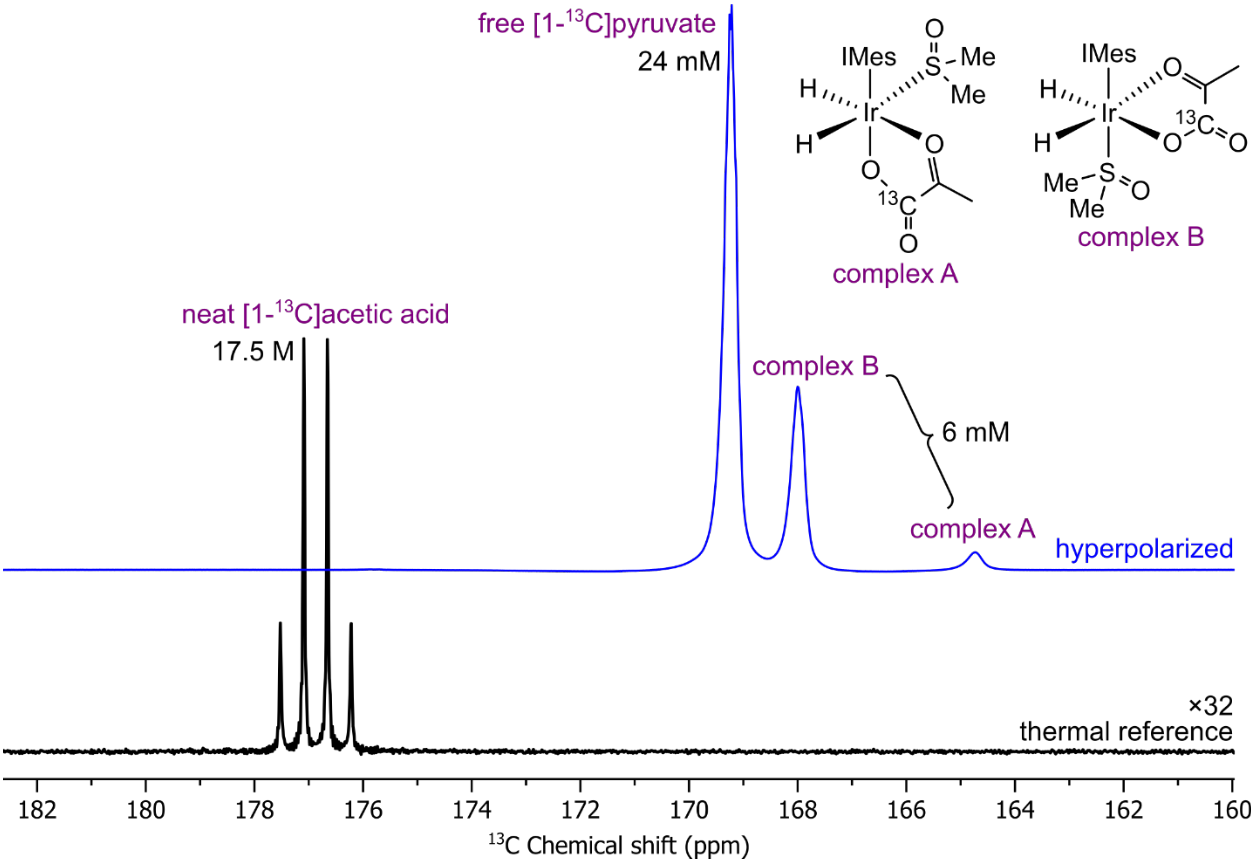

Figure S7. ^{13}C NMR spectra of SABRE-SHEATH-hyperpolarized [1-^{13}C]pyruvate (total concentration 30 mM, blue trace) and thermal reference spectrum of neat [1-^{13}C]acetic acid (17.5 M, black trace, multiplied by a factor of 32).

Table S1. [1-^{13}C]pyruvate SABRE-SHEATH polarization buildup data for the catalyst synthesized in this work (data for Figure 3A).

	Buildup time (s)	Integral	Signal enhancement	Polarization (%)
	5	136.153	25564.14401	3.09
	10	271.024	50887.57917	6.15
	15	390.669	73352.17422	8.86
	20	392.748	73742.52813	8.91
	25	435.337	81739.05651	9.87
	30	458.754	86135.84219	10.41
	35	516.988	97069.88229	11.73
	40	508.656	95505.4625	11.54
	45	518.22	97301.20313	11.75
	50	523.874	98362.80052	11.88
	55	526.459	98848.1612	11.94
	60	535.181	100485.8076	12.14
	65	525.236	98618.53021	11.91
	70	500.952	94058.95625	11.36
	75	526.848	98921.2	11.95
	80	543.056	101964.4208	12.32
	85	533.775	100221.8164	12.11
	90	544.211	102181.2841	12.34

Table S2. [1-^{13}C]pyruvate SABRE-SHEATH polarization buildup data for the catalyst made by literature procedure [1] (data for Figure 3B).

	Buildup time (s)	Integral	Signal enhancement	Polarization (%)
	5	112.48	21119.2917	2.55
	10	235.087	44140.0331	5.33
	15	333.025	62528.9128	7.55
	20	380.802	71499.5422	8.64
	25	367.331	68970.2216	8.33
	30	433.143	81327.1102	9.82
	35	440.681	82742.4482	10.00
	40	487.305	91496.5898	11.05
	45	482.564	90606.4177	10.95
	50	498.527	93603.6372	11.31
	55	511.549	96048.6534	11.60
	60	512.035	96139.9049	11.61
	65	513.038	96328.2286	11.64
	70	523.079	98213.531	11.86

	75	524.557	98491.0409	11.90
	80	528.382	99209.2245	11.98
	85	531.021	99704.7242	12.04
	90	530.246	99559.2099	12.03

Table S3. [1-^{13}C]pyruvate SABRE-SHEATH polarization decay data for the catalyst synthesized in this work (data for Figure 3C).

	**Decay time (s)**	**Integral**	**Signal enhancement**	**Polarization (%)**
	3	484.293	90931.0555	10.98
	5	484.772	91020.9927	11.00
	10	386.14	72501.8073	8.76
	15	285.288	53565.7938	6.47
	20	242.748	45578.4656	5.51
	25	192.236	36094.3115	4.36
	30	160.775	30187.181	3.65
	35	128.194	24069.7589	2.91
	40	119.401	22418.7815	2.71
	45	96.108	18045.2781	2.18
	50	80.179	15054.4424	1.82
	55	73.647	13827.9914	1.67
	60	57.628	10820.2573	1.31
	65	51.446	9659.5224	1.17
	70	47.315	8883.88411	1.07

Table S4. [1-^{13}C]pyruvate SABRE-SHEATH polarization decay data for the catalyst made by literature procedure [1] (data for Figure 3D).

	**Decay time (s)**	**Integral**	**Signal enhancement**	**Polarization (%)**
	3	463.257	86981.32734	10.51
	5	398.668	74854.06979	9.04
	10	395.387	74238.02786	8.97
	15	308.472	57918.83125	7.00
	20	261.325	49066.49089	5.93
	25	212.961	39985.64609	4.83
	30	174.351	32736.21641	3.95
	35	154.7	29046.53646	3.51
	40	133.692	25102.06563	3.03
	45	102.044	19159.82396	2.31
	50	107.699	20221.60911	2.44

	55	90.395	16972.60286	2.05
	60	83.705	15716.48568	1.90
	65	64.641	12137.02109	1.47
	70	59.42	11156.72396	1.35

References

1. Kownacki, I.; Kubicki, M.; Szubert, K.; Marciniec, B. Synthesis, Structure and Catalytic Activity of the First Iridium(I) Siloxide versus Chloride Complexes with 1,3-Mesitylimidazolin-2-Ylidene Ligand. *J. Organomet. Chem.* **2008**, *693*, 321–328. https://doi.org/10.1016/j.jorganchem.2007.11.013.
